# Mosquito Nets Treated with a Mixture of Chlorfenapyr and Alphacypermethrin Control Pyrethroid Resistant *Anopheles gambiae* and *Culex quinquefasciatus* Mosquitoes in West Africa

**DOI:** 10.1371/journal.pone.0087710

**Published:** 2014-02-03

**Authors:** Raphael N'Guessan, Corine Ngufor, Andreas A. Kudom, Pelagie Boko, Abibathou Odjo, David Malone, Mark Rowland

**Affiliations:** 1 Centre de Recherche Entomologique de Cotonou, Cotonou, Benin; 2 London School of Hygiene and Tropical Medicine, London, United Kingdom; 3 Pan-African Malaria Vector Research Consortium (PAMVERC), London, United Kingdom; 4 Department of Entomology and Wildlife, School of Biological Sciences, University of Cape Coast, Cape Coast, Ghana; 5 Innovative Vector Control Consortium, Liverpool, United Kingdom; Johns Hopkins Bloomberg School of Public Health, United States of America

## Abstract

**Background:**

The effectiveness of insecticide treated nets is under threat across Africa south of the Sahara from the selection of pyrethroid resistance in *Anopheles gambiae* mosquitoes. To maintain progress against malaria it is necessary to identify alternative residual insecticides for mosquito nets. Mixtures of pyrethroid and insecticides with novel mode of action provide scope for both improved control and management of resistance through concurrent exposure to unrelated insecticides.

**Methods:**

The pyrrole chlorfenapyr and the pyrethroid alphacypermethrin were tested individually and as a mixture on mosquito nets in an experimental hut trial in southern Benin against pyrethroid resistant *An gambiae* and *Culex quinquefasciatus* mosquitoes. The nets were deliberately holed to simulate the effect of wear and tear.

**Results:**

The nets treated with the mixture of chlorfenapyr 200 mg/m^2^ and alphacypermethrin 25 mg/m^2^ killed a proportion of *An gambiae* (77%, 95%CI: 66–86%) significantly greater than nets treated with alphacypermethrin 25 mg/m^2^ (30%, 95%CI: 21–41%) but not significantly different from nets treated with chlorfenapyr 200 mg/m^2^ (69%, 95%CI: 57–78%). The nets treated with the mixtures procured personal protection against *An gambiae* biting(58–62%) by a greater margin than the alphacypermethrin treated net (39%), whereas the chlorfenapyr treated net was not protective. A similar trend in mortality and blood feeding inhibition between treatments was observed in *Cx quinquefasciatus* to that seen in *An. gambiae*, although the effects were lower. A mixture of alphacypermethrin with chlorfenapyr applied at 100 mg/m^2^ had an effect similar to the mixture with chlorfenapyr at 200 mg/m^2^.

**Conclusion:**

The effectiveness of ITNs against pyrethroid resistant mosquitoes was restored by the mixture: the alphacypermethrin component reduced human-vector contact while the chlorfenapyr controlled pyrethroid-resistant mosquitoes. The complementary action of these unrelated insecticides demonstrates that the combination on nets has potential for preventing malaria transmission in areas compromised by the spread of pyrethroid resistance.

## Introduction

Long lasting insecticidal nets (LLINs) are considered best practice for malaria vector control because they are effective, reliable, robust and relatively simple to deliver even in remote regions [1]. The recent reductions in malaria-associated morbidity and mortality across sub Saharan Africa is largely attributed to the massive roll out of LLINs during the last decade [1]–[2]. Owing to their low cost, longer residual activity and safety, the pyrethroids remain the ideal insecticides for treating LLINs [1], [3]. Unfortunately, resistance to pyrethroids is spreading fast across Africa south of the Sahara and has now been reported in malaria vectors in 27 countries [4].

While a negative epidemiological impact of pyrethroid resistance on malaria control has yet to be demonstrated unequivocally, an increasing number of reports have indicated that pyrethroid resistance is capable of undermining the effectiveness of LLINs [5]–[6]. The situation in southern Benin is particularly grave since the premier brands of LLIN give only limited personal protection and kill fewer mosquitoes in that region than in regions of susceptibility [5]. It is now recognised that if nothing is done and further selection of resistance to pyrethroids leads to failure of LLINs, the progress achieved so far in reducing the burden of malaria could be reversed [7].

The need to identify alternative insecticides that can circumvent resistance and preserve the effectiveness of LLINs has become critical. Some alternative insecticides (such as the organophosphates and carbamates) have been tested on nets [8]–[9]. These candidates show toxicity but generally lack the irritancy of pyrethroids, an important property for reducing mosquito biting rates and providing personal protection to users of insecticide treated nets (ITNs). Mosquito nets can be treated with a mixture of pyrethroid and non-pyrethroid insecticides to maximise insecticidal efficacy against pyrethroid resistant mosquitoes while providing a degree of excito-repellency. Such mixtures may also provide opportunity for insecticide resistance management because insect phenotypes which are not killed by one component due to resistance will be controlled by the other provided they are not resistant to both insecticides [10]–[11].

An alternative insecticide to pyrethroids that is presently under development is chlorfenapyr, a pyrrole which owing to its novel mode of action is active against both pyrethroid resistant and susceptible anophelines and culicines [12]–[13]. When evaluated on mosquito nets against wild mosquitoes in experimental huts, chlorfenapyr was found to be more effective than pyrethroids at killing resistant *Anopheles* and *Culex* [14]–[15]. The use of this pyrrole by itself on mosquito nets neither deters nor repels and so needs to be combined with an excito-repellent insecticide [15]. Chlorfenapyr has already shown great promise when applied as IRS in conjunction with LLINs for improved transmission control of pyrethroid resistant mosquitoes [16]. Preliminary studies with a mixture of chlorfenapyr and alphacypermethrin on ITNs has shown good potential for control of *An arabiensis* [17].

The objective of the trial reported here was to determine in experimental huts the potential of mosquito nets treated with a mixture of chlorfenapyr and the pyrethroid alphacypermethrin to protect against and control host-seeking *An gambiae* and *Cx quinquefasciatus* that are strongly resistant to pyrethroids.

## Materials and Methods

### Study site and experimental huts

The study was conducted on a private land at Akron, a village on the outskirts of Porto Novo, Benin. The owner of the land gave permission to conduct the study on this site. The site supported breeding of *An gambiae* M form that are pyrethroid-resistant due to high frequency of *kdr* (>90%) and increased activity of cytochrome P450s [18]. The nuisance mosquito *Cx quinquefasciatus* is present year round and shows resistance to pyrethroids, carbamates and organophosphates [19]. Five experimental huts were selected for the trial. The hut design was the West African type recommended by WHO [20].

### Mosquito net treatments and trial procedure

The nets were made of white 100-denier polyester (SiamDutch Mosquito Netting Co., Bangkok, Thailand). To simulate damaged nets, six standardized holes (each measuring 4 cm×4 cm) were cut into the sides and ends of each net as recommended by WHO [20]. The nets were treated with SC formulations of alphacypermethrin (Fendona 60SC, BASF, Germany) and chlorfenapyr (Phantom 240SC, BASF, Germany) in aqueous solution either separately or mixed together. The treatment arms were: (i) untreated control net, (ii) chlorfenapyr 200 mg/m^2^ treated net, (iii) alphacypermethrin 25 mg/m^2^ treated net, (iv) chlorfenapyr 100 mg/m^2^ and alphacypermethrin 25 mg/m^2^ mixture treated net, and (v) chlorfenapyr 200 mg/m^2^ and alphacypermethrin 25 mg/m^2^ mixture treated net.

The 5 treatments were randomly allocated to the 5 experimental huts and rotated weekly between huts. Adult volunteers slept in the huts from 20.00 - 05.00 and were rotated between huts on consecutive nights. Informed consent to participate was given before recruitment and daily chemoprophylaxis was provided until 4 weeks after the trial. Each morning the volunteers helped to collect mosquitoes from inside the rooms, nets and verandah traps. Mosquito collections were made on 36 nights over six weeks between April and June 2010. The identification to species, mortality counts and determination of feeding status and gonotrophic condition were made in the laboratory. Live mosquitoes were held in netted plastic cups and provided with 10% honey solution; mortality was recorded after 72 h. Climatic information for each day of the trial was collected from the weather station based in Porto Novo.

The principal aim was to assess the efficacy of treatments relative to untreated control nets in terms of (i) deterrency: the proportional reduction in the number of mosquitoes entering huts with treated nets, (ii) insecticide induced exiting rates: estimated from the proportions of mosquitoes collected from the verandahs of treatment and control huts, (iii) blood-feeding inhibition rate: the reduction in the proportion of blood fed mosquitoes in huts with treated nets compared to huts with untreated nets = (1−proportion bloodfed in treatment**/**proportion bloodfed in control)×100; (iv) mortality rate: the proportion of mosquitoes dying within 72 h, corrected for control mortality; (v) personal protection.

### Data analysis

The analysis of numbers of mosquitoes collected within huts (overall total, blood-fed and dead totals) was carried out using negative binomial regression after adjusting for the effects of hut and sleeper. The analysis of treatment effects on the proportions of mosquitoes blood-feeding, exiting or killed was carried out using logistic regression, with treatments as fixed effects and huts, sleepers as random effects. All statistical analysis was conducted by using STATA 9 software (STATA Corp., College Station, USA).

### Species and resistance characterisation

Samples of *An. gambiae* s.l. reared from larval collections near the trial site were identified to species using PCR [21] and to molecular form using PCR RFLP [22].

WHO test kits, lined with test papers treated with alphacypermethrin in silicon oil were used to determine susceptibility of *An. gambiae* and *Cx. quinquefasciatus* females reared from larval collections.

PCR diagnostic test for detection of *kdr* mutations was carried out on *An. gambiae* and *Cx. quinquefasciatus* mosquitoes as described by Martinez-Torres et al. [23].

### Toxicology

Chlorfenapyr has a WHO toxicological classification III, an LD50 oral toxicity in rats of >400 mg/kg body weight and acute dermal toxicity >2000 mg/kg; a category of similar to many insecticides used in public health [24]. A risk assessment of the use of chlorfenapyr on nets was undertaken by BASF toxicologists using assumptions, parameters and default values defined in the WHO generic risk assessment model [25]. The calculated exposure levels to chlorfenapyr were all below the relevant dermal and systemic acceptable exposure levels for repeated exposure. Exposure was deemed acceptable based on conservative scenarios from the WHO model, indicating safe use of the chlorfenapyr-treated nets for the intended use.

### Ethical clearance

Written informed consent was obtained from all volunteers recruited to the experimental hut studies. The study was approved by ethics committees of the London School of Hygiene and Tropical Medicine and the Ministry of Health in Benin.

## Results

### Experimental Hut Trial

Over the six week trial, 515 *Anopheles gambiae* s.l., 3764 *Culex quinquefasciatus* and 453 *Mansonia* females were caught in the huts. Only the data on *An. gambiae* s.l. and *Cx. quinquefasciatus* were analysed further (Table 1&2).

**Table 1:** Experimental hut trial results against pyrethroid resistant *Anopheles gambiae*.

	Untreated net	Chlorfenapyr 200 mg/m^2^	Alphacypermethrin 25 mg/m^2^	Mixture 1 (Chlorfenapyr 100+Alpha 25)	Mixture 2 (Chlorfenapyr 200+Alpha 25)
Total females caught	102^a^	76^b^	81^b^	80^b^	66^b^
Deterrence %	-	26	21	22	35
Total females in verandah trap	36	31	56	59	50
Exiting % (95% CI)	35 (26–45)^a^	41 (30–52)^a^	69 (59–79)^b^	74 (64–83)^b^	76 (65–86)^b^
Total females blood fed	77	59	47	29	32
Blood-feeding % (95% CI)	75 (66–83)^a^	78 (68–87)^a^	58 (47–69)^b^	36 (26–47)^c^	49 (36–61)^bc^
Personal protection %	-	23	39	62	58

Mixture 1 and 2 applied alphacypermethrin at 25 mg/m^2^ and chlorfenapyr at 100 and 200 mg/m^2^ respectively. Numbers in the same row sharing a letter superscript do not differ significantly (P>0.05).

**Table 2:** Experimental hut trial results against pyrethroid resistant *Culex quinquefasciatus*.

	Untreated net	Chlorfenapyr 200 mg/m2	Alphacypermethrin 25 mg/m2	Mixture 1 (Chlorfenapyr 100+Alpha 25)	Mixture 2 (Chlorfenapyr 200+Alpha 25)
Total females caught	811^a^	527^b^	567^b^	487^b^	492^b^
Deterrence %	-	35	30	40	39
Total females in verandah trap	227	279	408	370	354
Exiting % (95% CI)	28 (25–31)^a^	53 (49–57)^b^	72 (68–75)^c^	76 (72–79)^c^	72 (68–76)^c^
Total females blood fed	430	200	57	44	59
Blood-feeding % (95% CI)	53 (49–56)^a^	38(34–42)^b^	10 (7–12)^c^	9 (7–12)^c^	12 (7–15)^c^
Personal protection %	-	53	87	90	86

Mixture 1 and 2 applied alphacypermethrin at 25 mg/m^2^ and chlorfenapyr at 100 and 200 mg/m^2^ respectively. Numbers in the same row sharing a letter superscript do not differ significantly (P>0.05).

Compared to huts containing untreated nets the entry rates of both species into huts with ITNs were up to 40% less (p<0.001) (Table 1&2). Hut entry rates did not differ significantly between huts with alphacypermethrin, chlorfenapyr or mixture treated nets.

In huts where nets were treated with alphacypermethrin or alphacypermethrin-chlorfenapyr mixtures the proportions of *An. gambiae* and *Cx. quinquefasciatus* that exited into verandahs were significantly greater than huts where nets were untreated or treated with chlorfenapyr (p<0.0001) (Table 1&2). For *An. gambiae* the proportions that exited into verandahs did not differ between huts with chlorfenapyr treated or untreated nets (Table 1), whereas for *Cx. quinquefasciatus* the proportion that exited into verandahs was greater for chlorfenapyr treated nets (p<0.001) (Table 2).

**Figure 1:**
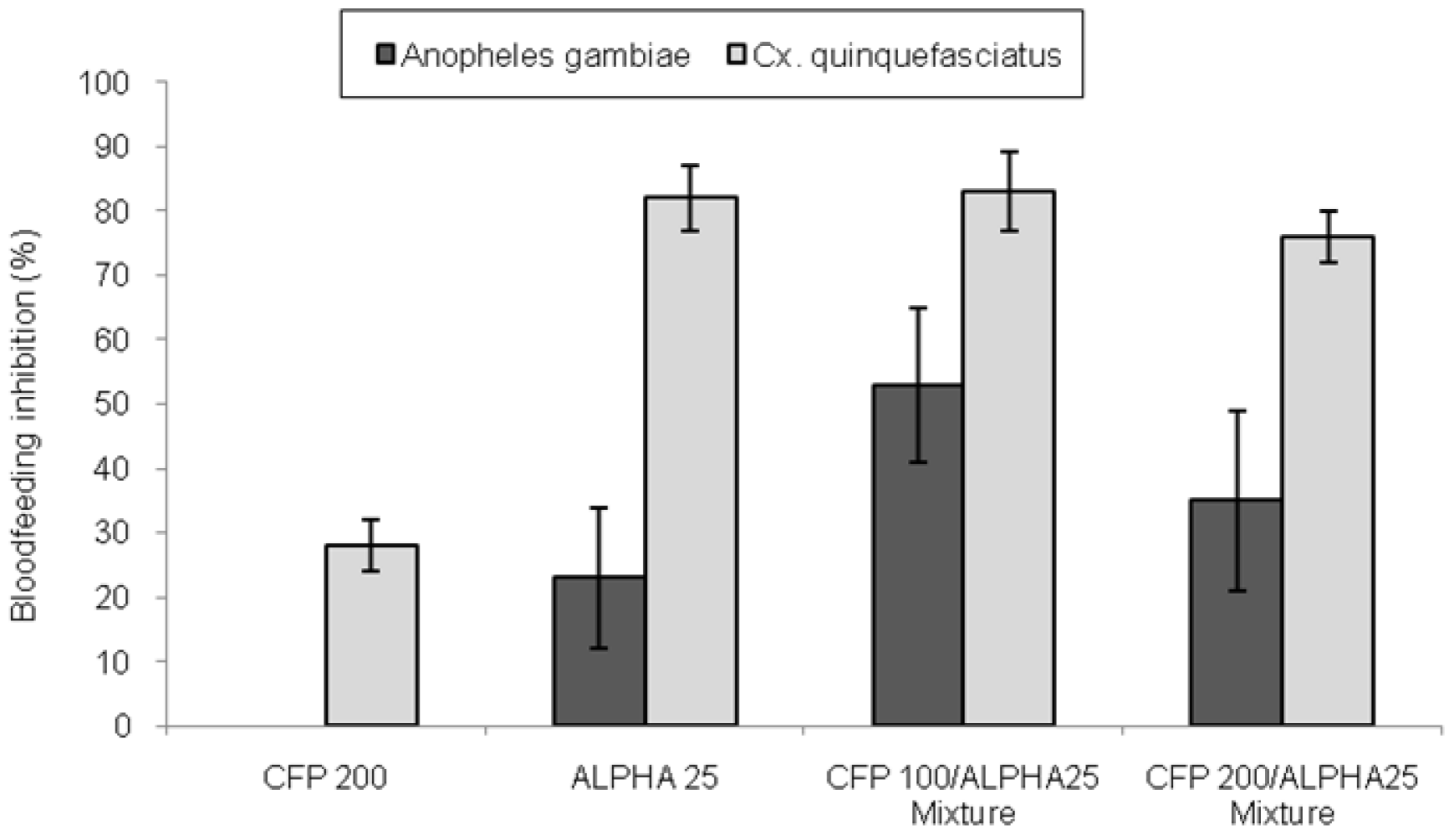
Blood-feeding inhibition rates of *Anopheles gambiae* and *Culex quinquefasciatus* in experimental huts.

Blood-feeding inhibition of *An. gambiae* was not evident where nets were treated with chlorfenapyr alone (figure 1A). There was some blood-feeding inhibition of *An. gambiae* where nets were treated with alphacypermethrin alone (22%, P = 0.042). and further inhibition where nets were treated with the mixtures (35%, 51%, P<0.01). The difference in bloodfeeding inhibition between the mixture with chlorfenapyr 200 mg/m2 and alphacypermethrin alone was not significant (P = 0.061).

Blood-feeding inhibition rates were generally higher for *Cx. quinquefasciatus* than for *An. gambiae*, reaching 28% inhibition for the chlorfenapyr net, 81% for the alphacypermethrin net and 78% and 82% for the mixtures (Figure 1). For *Cx. quinquefasciatus* the inhibition of blood-feeding was attributable more to the pyrethroid component than to the chlorfenapyr component.

Personal protection against the biting of *An gambiae* ranged from 58% to 62% with the insecticide mixtures, higher than with either insecticide when applied to nets alone (Table 1). Personal protection from the mixtures was higher still against *Cx. quinquefasciatus* (86% to 90%) but was not significantly different from alphacypermethrin net (Table 2).

**Figure 2:**
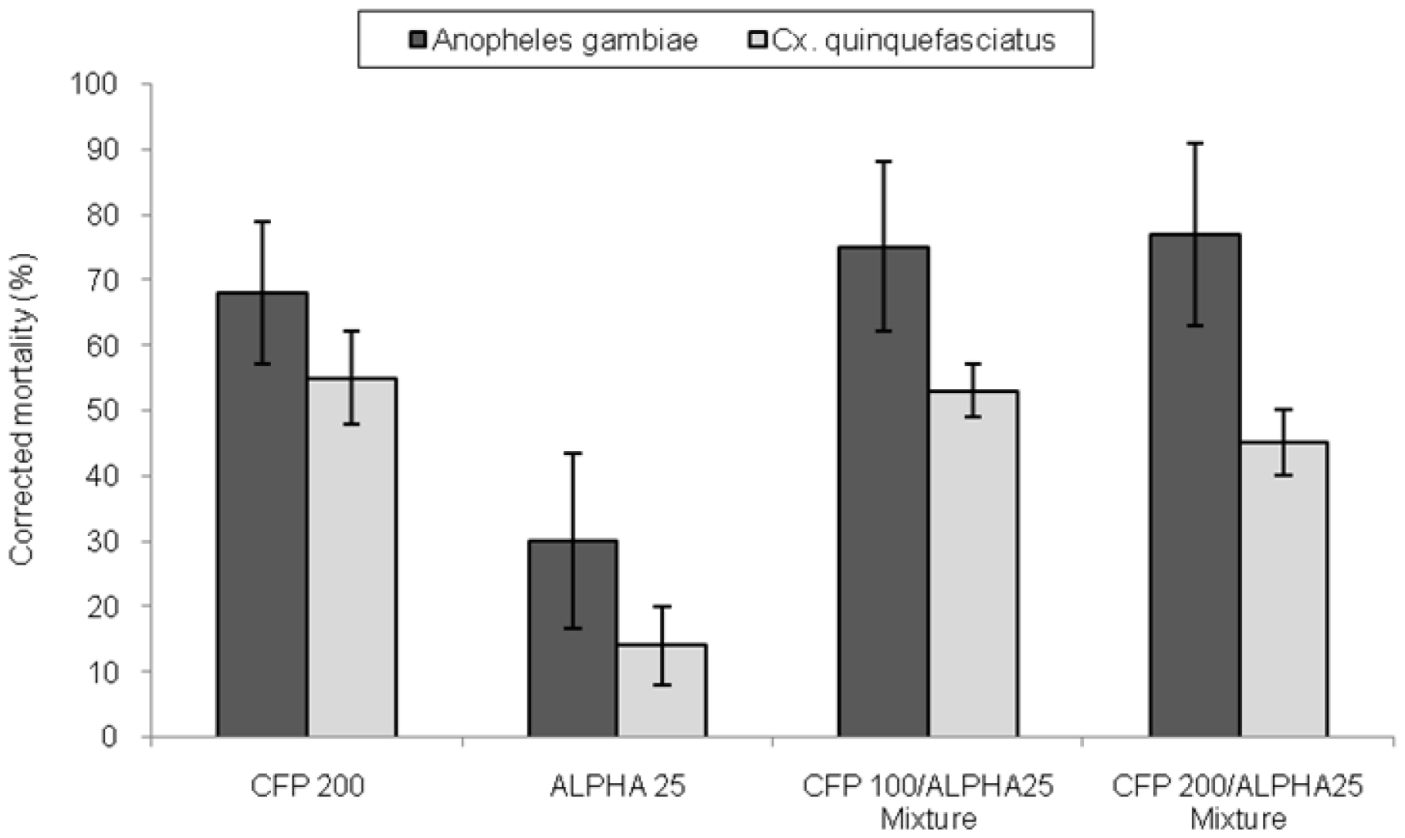
Control corrected mortality of *Anopheles gambiae* and *Culex quinquefasciatus* in experimental huts.

Mortality rates of *An. gambiae* and *Cx. quinquefasciatus* where nets were treated with alphacypermethrin were less than 30%, and were presumably due to the high level of pyrethroid resistance in the two species (Figure 2). Nets treated with the insecticide mixtures induced three times higher mortality of *An. gambiae* (75%) and *Cx quinquefasciatus* (47–50%) than the alphacypermethrin treated nets (P<0.01). Mortalities of *An. gambiae* with the mixtures were not significantly different to that induced by chlorfenapyr alone (P = 0.082 for the mixture with chlorfenapyr 100 mg/m^2^ and P = 0.065 for chlorfenapyr 200 mg/m^2^) and hence the mixture's toxicity is attributable mainly to the chlorfenapyr component (Figure 2). The difference in mortality of *An. gambiae* between mixtures with low and high dosages of chlorfenapyr was not significant (P = 0.917).

A similar trend in mortality to that seen in *An. gambiae* was observed in *Cx quinquefasciatus*, although the effect of each treatment on mortality was 20–50% lower.

The average daily temperature (minimum, minimum) during the first week of the trial was 31.5 degree (27.7, 34.3). Temperature gradually decreased each week and by the 12^th^ week was 27.8 (25.4, 30.2).

### Residual activity

Cone bioassays conducted each month with the *An. gambiae* (Kisumu) susceptible strain on nets treated with the mixture or alphacypermethrin induced 87–100% mortality (N = 50 per test) throughout the 3 months. Bioassays on nets treated with chlorfenapyr killed 77% initially and 65% by the end of the trial.

### Species characterization and resistance status

Only *An. gambiae* s.s. M form was found in the trial area. In WHO susceptibility tests with pyrethroid test papers percentage mortality was 20% for *An gambiae* and 17% for *Cx quinquefasciatus*. In molecular assays on *An. gambiae* the frequency of *kdr* was 0.86. No molecular assays were conducted on contemporary samples of *Cx quinquefasciatus* but in an earlier characterization in the area the frequency of *kdr* was 0.63 [19].

## Discussion

The aim of the study was to determine whether ITNs treated with a mixture of alphacypermethrin and chlorfenapyr have the potential to provide individual protection against mosquito biting and control of mosquitoes in regions of West Africa where the development of pyrethroid resistance in *An. gambiae* is undermining the effectiveness of pyrethroid treated nets and threatening malaria control. In experimental huts nets treated with the insecticide mixture reduced survival of *An. gambiae* by 75% and prevented host-vector contact by 40–50%, an effect similar to pyrethroid treated nets and LLINs in areas that have not yet developed pyrethroid resistance [5], [26]. The loss of efficacy of pyrethroid treated nets when encountering with pyrethroid resistance at the level that occurs in Southern Benin [6], [27] was therefore restored by the mixture.

By combining a pyrethroid and a pyrrole on the same net, it was possible to benefit from the properties of each insecticide: the protective (excito-repellent) effect of the pyrethroid and the killing effect of the pyrrole against pyrethroid resistant Anopheline and Culicine mosquitoes. The low rates of mosquito mortality and blood feeding inhibition shown by the alphacypermethrin ITN being compared were typical of pyrethroid ITN trials in this area against *An. gambiae* and *Cx. quinquefasciatus* [5], [27]. Transmission control through mosquito mortality and personal protection from mosquito biting are important attributes of any vector control tool as these work together to reduce vectorial capacity. By both procuring individual protection and killing mosquitoes the chlorfenapyr-alphacypermethrin mixture shows greater potential for malaria control in areas with high level pyrethroid resistance than could be achieved by pyrethroid LLINs.

Chlorfenapyr toxicity to mosquitoes in bioassay is positively temperature dependent (N'Guessan and Rowland, unpublished data). At the ambient temperatures recorded outdoors during the trial the lowest minimum daily temperature was 25.4 and the highest minimum daily temperature was 28.7 degrees. Chlorfenapyr was very effective within this range.

Previously, mixtures of a carbamate (carbosulfan) and a pyrethroid were evaluated on mosquito nets but were not taken forward due to mammalian toxicity issues associated with the carbamate [28]. Chlorfenapyr has a non neurological mode of action and would be a more appropriate companion insecticide due to its safety [24], effectiveness against mosquitoes, and absence of cross resistance to any existing class of insecticide [13], [29]. Reducing the concentration of chlorfenapyr by half (to 100 mg/m^2^) did not reduce the effectiveness of the mixture, and has obvious cost benefits.

Quite apart from the restoration of control which mixtures promise, empirical research and modelling predict that mixtures are the most efficient tactic for managing insecticide resistance [7]. The attributes required of mixtures in order to prevent the selection of resistance alleles are rigorous, and include the maintenance of effective concentrations of both insecticide components over the lifetime of the net [10]–[11]. This is the challenge which the next generation of bi-treated LLINs and the formulators of mixtures on nets must meet. In any event, insecticide mixtures for malaria control are a modern day reality. LLIN coverage is going universal and IRS with non-pyrethroid insecticides is being applied concurrently with LLINs as malaria control policy in many areas of high malaria transmission [30]–[31]. Chlorfenapyr is already showing potential as an IRS treatment in combination with pyrethoid LLIN [16]. A long lasting mixture of chlorfenapyr and alphacypermethrin on nets, if realised, will make an essential contribution to the next generation of LLINs and to prevention of malaria.

## Supporting Information

Table S1 Checklist of information when reporting an experimental hut trial.
(DOC)
